# Selenium-Enriched Yeast Alleviates Oxidative Stress-Induced Intestinal Mucosa Disruption in Weaned Pigs

**DOI:** 10.1155/2020/5490743

**Published:** 2020-03-19

**Authors:** Lei Liu, Caimei Wu, Daiwen Chen, Bing Yu, Zhiqing Huang, Yuheng Luo, Ping Zheng, Xiangbing Mao, Jie Yu, Junqiu Luo, Hui Yan, Jun He

**Affiliations:** ^1^Institute of Animal Nutrition, Sichuan Agricultural University, Sichuan Province, Chengdu 611130, China; ^2^Key Laboratory of Animal Disease-Resistant Nutrition, Sichuan Province, Chengdu 611130, China

## Abstract

To explore the effect of selenium-enriched yeast (SeY) on intestinal barrier functions in weaned pigs upon oxidative stress, a 2 × 2 factorial design was utilized and thirty-two pigs were randomly assigned into four groups. Pigs with or without exposure to oxidative stress (diquat challenge) were fed with a basal diet or a SeY-containing diet. The trial lasted for 21 days, and result showed that SeY supplementation attenuated body-weight reduction and significantly decreased the serum concentrations of diamine oxidase (DAO) and D-lactic acid in pigs upon diquat challenge (*P* < 0.05). Diquat challenge decreased the villus height and the ratio of villus height to crypt depth (V/C) in the jejunum and ileum (*P* < 0.05). However, SeY supplementation not only elevated the villus height and the ratio of V/C (*P* < 0.05) but also improved the distribution and abundance of tight-junction protein ZO-1 in the jejunum epithelium. Interestingly, SeY supplementation acutely decreased the total apoptosis rate of intestinal epithelial cells in pigs upon diquat challenge (*P* < 0.05). Moreover, SeY elevated the content of antioxidant molecules such as glutathione peroxidase (GSH-Px) and catalase (CAT) but significantly decreased the content of malondialdehyde (MDA) in the intestinal mucosa (*P* < 0.05). Importantly, SeY elevated the expression levels of critical functional genes such as the nuclear factor erythroid-2-related factor 2 (Nrf2), heme oxygenase-1 (HO-1), sodium/glucose cotransporter 1 (SGLT1), and B-cell lymphoma-2 (BCL-2) in the intestinal mucosa upon diquat challenge (*P* < 0.05). Moreover, the expression of caspase-3 was downregulated by SeY in the duodenum and jejunum mucosa (*P* < 0.05). These results indicated that SeY attenuated oxidative stress-induced intestinal mucosa disruption, which was associated with elevated mucosal antioxidative capacity and improved intestinal barrier functions.

## 1. Introduction

Intestinal barrier mainly consists of a single layer of enterocytes and intercellular tight junctions of enterocytes. It serves as a selective permeable membrane that is not only responsible for nutrient digestion and absorption but also constitutes the first line of defence to prevent a variety of harmful substances from entering the intestinal mucosa and systemic circulation [1]. Moreover, the gastrointestinal tract also serves as the biggest immune organ for animals [2]. A variety of stimuli such as bacterial infections and stresses may impair the integrity of the intestinal barrier. For instance, overproduction of reactive oxygen species (ROS) not only promoted secretion of inflammatory cytokines but also induced intestinal mucosal damage and dysfunction [3]. Evidence is accumulating to show that overproduction of ROS may result in DNA damage and intestinal cell apoptosis, which increases the intestinal permeability and facilitates translocation of luminal antigens into subepithelial tissues leading to a series of intestinal and systemic inflammatory response [4]. Therefore, the avenue to alleviate the ROS-induced disruption of the intestinal barrier has attracted considerable research interest worldwide.

Selenium (Se) is an essential trace element for animals. Importantly, Se is a critical component of the enzyme glutathione peroxidase (GSH-Px), which detoxifies lipid peroxides and provides protection of cellular and subcellular membranes against ROS damage [5]. As compared to inorganic forms of Se (i.e., sodium selenite and sodium selenate), the Se-enriched yeast (SeY) is mainly consisted of selenoamino acids (i.e., selenomethionine) and their analogues [6]. Previous studies indicated that the SeY was more biologically available than inorganic forms [7]. Importantly, the SeY was found to reduce ROS production and cellular oxidative damage in a variety of animal species [8, 9]. Moreover, the SeY can also act as a clinical health product or drug for various diseases such as Alzheimer's disease [10].

Weaning is a critical developmental stage for neonatal mammals. However, neonatal mammals are more susceptible to various stresses (i.e., oxidative stress) at the weaning stage than other stages because of changes of their living conditions and transition of liquid feed to solid feed. A previous study has indicated that oxidative stress is one of the major causes leading to enterocyte apoptosis and cell cycle arrest in weaning pigs [11]. Pig is an excellent model species used in biomedical researches, as they are closely related to humans in terms of anatomy, genetics, and physiology. Moreover, both species are omnivorous and their organs generally share common functional features [12]. In the present study, we investigated the effect of dietary SeY supplementation on antioxidative capacity and intestinal health in weaned pigs upon oxidative stress. Moreover, the mechanism behind the SeY-modulated intestinal barrier functions has also been explored. This study could also assist in developing of potential clinical health products or drugs for the ROS-induced diseases.

## 2. Materials and Methods

### 2.1. Materials

The oxidative stress was induced using diquat injection as described by previous studies [1, 13, 14]. Diquat was purchased from Shanghai Yuanye Biotechnology Co., Ltd., with an average molecular weight of 362.06 and purity of 99%. 10 mg/kg of diquat solution was prepared with sterilized physiological saline and stored at 4-8°C (Shanghai, China) until its use. The SeY was purchased from Sichuan Junzheng Biofeed Co., LTD (organic selenium ≥ 95%).

### 2.2. Animals and Study Design

Thirty-two weaned pigs (with an average initial body weight 7.30 ± 0.14 kg) were randomly assigned into four treatments consisting of CON (pigs fed with basal diet), SSY (pigs fed with basal diet containing 250 mg/kg SeY), DT (pigs fed with basal diet), and DSY (pigs fed with basal diet containing 250 mg/kg SeY). The trial lasted for 21 d. On 16 d, the CON and SSY groups received intraperitoneal injection of sterile saline (0.9%), whereas the DT and DSY groups received injection of diquat (10 mg/kg BW). All procedures used in the animal experiment were approved by the Institutional Animal Care and Use Committee of Sichuan Agricultural University (no. 20181105). The basal diet (Table 1) was formulated to meet the nutrient requirements recommended by the National Research Council 2012 [15]. Pigs were individually housed in metabolism cages (0.7 m × 1.5 m) and were given ad libitum access to fresh water and feed.

### 2.3. Sample Collection and Preparation

At the end of the trial, blood samples were collected by venepuncture at 8:00 after 12 h of fasting. Then, the samples were centrifuged at 3500 × g at 4°C for 10 min. After centrifugation, the serum samples were collected and frozen at −20°C until analysis. After blood collection, pigs were euthanized with an intravenous injection of sodium pentobarbital and then slaughtered by exsanguination protocols. Sections of the duodenum, jejunum, and ileum were immediately isolated. Approximately 5 cm segments of the middle of duodenum, jejunum, and ileum were gently flushed with ice-cold phosphate-buffered saline (PBS) and then fixed in 4% paraformaldehyde solution for morphological analyses and immunofluorescence. Finally, the residual duodenal, jejunal, and ileal segments were scraped with a scalpel blade, and the mucosa samples were collected and stored at −80°C until analysis.

### 2.4. Serum Biochemical Analysis

The concentrations of D-lactic acid and the activity of diamine oxidase (DAO) in serum were determined using commercially available swine Enzyme-Linked Immunosorbent Assay (ELISA) kits (Jiangsu Jingmei Biotechnology Co., Ltd., Yancheng, China).

### 2.5. Intestinal Morphology Analysis

About 1 cm segment of the small intestine (duodenum, jejunum, and ileum) was mixed in 10% neutral buffered formaldehyde. The mixed tissue samples were dehydrated with normal saline and then embedded in paraffin. Cross sections of each sample were prepared, stained with haematoxylin and eosin (H&E), and then sealed by a neutral resin size. Ultrathin sections of the duodenal, jejunal, and ileal samples were examined for the villus height and crypt depth with image processing and analysis system (Media Cybernetics, Bethesda, MD, USA). Villus height was calculated from the tip of the villi to the villus-crypt junction. Crypt depth was expressed as the invaginated depth between adjacent villi. A total of 10 intact, well-oriented crypt-villus units were analysed in triplicate per segment. The ratio of villus height to crypt depth (V/C) was calculated from the values described above.

### 2.6. Immunofluorescence Analysis

The localization of ZO-1 protein in jejunal tissues was determined by immunofluorescence as in the previous method. 4% paraformaldehyde-fixed samples were rinsed in PBS and then incubated with ethylenediaminetetraacetic acid (EDTA, 1 mol/L, pH 9.0, Gooddbio Technology Co., Ltd., Wuhan, China) for antigen retrieval. Tissue sections were blocked with 3% bovine serum albumin prior to incubation with rabbit anti-ZO-1 polyclonal antibody (1 : 250; Abcam Plc., Cambridge, UK) overnight at 4°C. Slides were then washed three times with PBS and incubated with goat anti-rabbit IgG-FITC secondary antibody (Gooddbio Technology Co., Ltd., Wuhan, China) for 1 h at room temperature in the dark. Finally, slides were washed three times with PBS, and the nuclei were stained with 4′-6-diamidino-2-phenylindole (DAPI, Gooddbio Technology Co., Ltd., Wuhan, China) for 10 min at room temperature in the dark. All slides were examined for fluorescence using a confocal scanning microscope (Nikon Eclipse TI-SR), and images were taken with the NIKON DS-U3 software.

### 2.7. Flow Cytometry Assays

The percent of jejunum cell apoptosis was determined by flow cytometry. Remove intestinal tissue from the centrifugal tube and flush gently by ice-cold phosphate-buffered saline (PBS: for 1 liter: 8.00 g NaCl, 0.20 g KCl, 1.78 g Na_2_HPO_4_·2 H_2_O, 0.27 g KH_2_PO_4_, pH 7.4), apply the washed intestinal serosa layer to the ice pack, scrape the mucosal cells with a glass slide, cut the tissue block with scissors in the sterile watch glass, add proper RPMI 1640 medium (HyClone, America), then transfer to a new centrifuge tube, and mix the cells on a vortex mixer. Filter the cells in a flow tube with a 300-mesh filter cloth, centrifuge at 300 × g for 5 min, discard the supernatant, and wash again with PBS. Resuspend cells with 200 *μ*L Binding Buffer (10 mM HEPES, 140 mM NaCl, 2.5 mM CaCl_2_, pH 7.4) and adjust the cell concentration to 10^6^ cells/mL with PBS, 4°C. Take 100 *μ*L of cell suspension into a flow tube, resuspend the cells by adding 1 mL of Binding Buffer, centrifuge at 300 × g, 5 min, and discard the supernatant. Add 5 *μ*L Annexin V-FITC (Invitrogen, Australia) staining for fluorescence staining, 10 min, room temperature, and dark; apply 5 *μ*L of PI (propidium iodide) staining solution, 5 min, room temperature; and incubate 500 *μ*L of Binding Buffer. It was detected by CytoFLEX flow cytometry (Beckman, America) with CytExpert software (Beckman, America).

### 2.8. Enzyme Activity Assays

Several antioxidant-related parameters including catalase (CAT), glutathione (GSH), malondialdehyde (MDA), and total antioxidant capacity (T-AOC) were measured using the assay kits and associated protocols supplied by Nanjing Jiancheng Bioengineering Institute (Nanjing, China).

### 2.9. RNA Isolation, Reverse Transcription, and Real-Time Quantitative PCR

Total RNA was extracted from duodenum, jejunum, and ileum mucosa using the Trizol Reagent (TaKaRa, Dalian, China). Meanwhile, the concentration and purity of total RNA were assayed by a spectrophotometer (NanDrop, Gene Company Limited, Guangzhou, China) at 260 and 280 nm following the manufacturer's guidelines. The ratio of OD 260/280 should vary between 1.8 and 2.0. Reverse transcription using the PrimeScript RT reagent kit (TaKaRa Biotechnology, Dalian, China) was exploited following the manufacturer's instructions. The primers were synthesized commercially by Life Technologies Limited and was exhibited in Table 2.

Quantitative real-time polymerase chain reaction (PCR) was conducted to analyse the mRNA expression abundance of SGLT1, GLUT2, Bcl-2, caspase-3, Nrf2, and HO-1 in the small intestinal mucosa using the CFX-96 real-time PCR detection system (Bio-Rad) and SYBR Premix Ex Taq II (Tli RNaseH Plus) reagents (TaKaRa, Dalian, China). The PCR reaction was run in a 10 *μ*L reaction volume, which contained 5 *μ*L of SYBR Premix Ex Taq II (Tli RNaseH Plus), 0.5 *μ*L of each primer, 1 *μ*L of the cDNA sample, and 3 *μ*L of nuclease-free water. The PCR cycling parameters were as follows: initial denaturation at 95°C for 30 s, followed by 40 cycles of 95°C for 5 s, 57.5°C for 30 s, and 72°C for 5 min. A melting curve analysis was performed following each real-time quantitative PCR assay to confirm the gene-specific amplification products had been generated. The housekeeping gene *β*-actin was used as an internal control for normalization. The target gene mRNA expression level was calculated using the 2^−*ΔΔ*Ct^ method [16]. Each sample was simultaneously performed on the same PCR plate, and three replicates were set up.

### 2.10. Statistical Analysis

Data before the injection were analysed by one-way ANOVA. Data after the injection were analysed by two-way ANOVA using the general linear model procedure. Model main effects included SeY and oxidative stress (injection of diquat or saline). Probability values < 0.05 were considered to indicate a significant difference and values between 0.05 and 0.10 to indicate a trend. Variable means for treatments showing significant differences in the ANOVA were separated by Duncan's multiple-range test (*P* < 0.05). Values were expressed as means with their standard errors. All statistical analysis was performed using SPSS 24.0 (SPSS, Inc.).

## 3. Results

### 3.1. Effect of SeY on Growth Performance in Weaned Pigs upon Oxidative Stress

As shown in Table 3, there were no significant differences (*P* > 0.05) in average daily food intake (ADFI), average daily gain (ADG), and feed efficiency (G : F) among the four groups from d 1 to d 15 (preinjection). After diquat injection, the ADG and ADFI were significantly reduced in the DT and DSY groups, as compared to those in the CON and SSY groups (*P* < 0.05). However, the ADG and ADFI were higher in the DSY group than those in the DT group (*P* < 0.05).

### 3.2. Effect of SeY on Serum DAO and D-Lactic Acid Concentrations in Weaned Pigs upon Oxidative Stress

**Figure 1:**
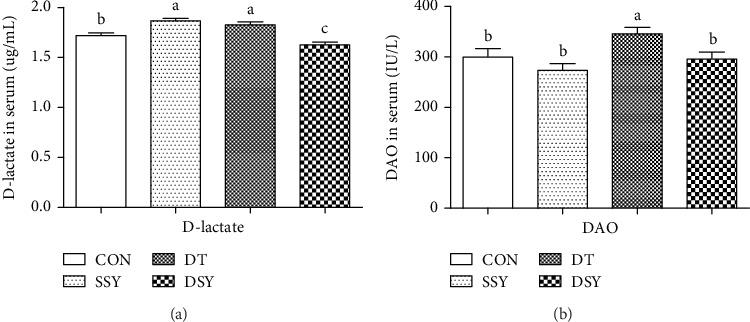
Effect of SeY on serum parameters in weaned pigs upon diquat challenge. ^a,b,c^Mean values with different letters on vertical bars indicate significant differences (*P* < 0.05); CON: pigs were fed with basal diet and challenged by sterile saline; SSY: pigs were fed with SeY-containing diet and challenged by sterile saline; DT: pigs were fed with basal diet and challenged by diquat; DSY: pigs were fed with SeY-containing diet and challenged by diquat.

As shown in Figure 1, the serum concentrations of DAO and D-lactic acid were significantly higher in the DT group than those in the CON group (*P* < 0.05). However, the serum concentrations of DAO and D-lactic acid were significantly decreased in the DSY group, as compared to those in the DT group (*P* < 0.05).

### 3.3. Effect of SeY on Intestinal Morphology and Distribution of the Tight-Junction Protein ZO-1

**Figure 2:**
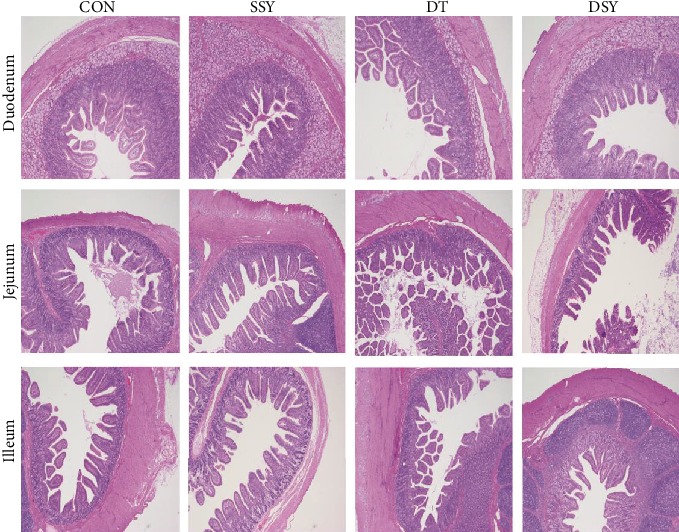
Effect of SeY on small intestinal morphology in weaned pigs (H&E; ×40). CON: pigs were fed with basal diet and challenged by sterile saline; SSY: pigs were fed with SeY-containing diet and challenged by sterile saline; DT: pigs were fed with basal diet and challenged by diquat; DSY: pigs were fed with SeY-containing diet and challenged by diquat.

**Figure 3:**
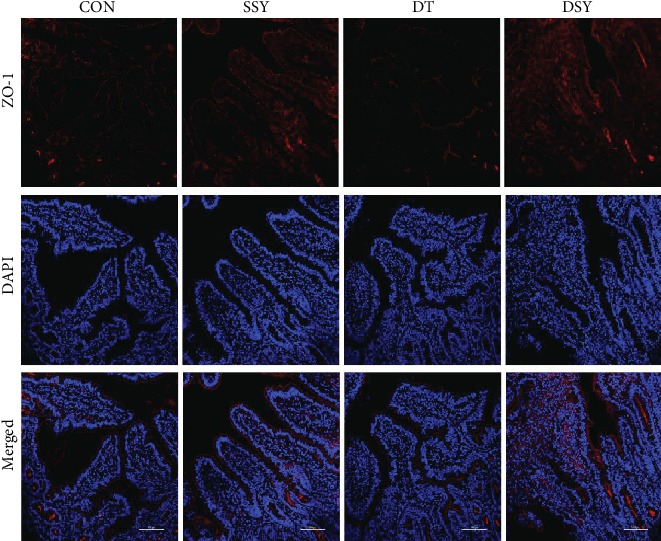
Effect of SeY on tight junction distribution and localization of ZO-1 in the intestinal epithelium. Localization of ZO-1 and DAPI (DNA) within the jejunum-weaned pigs was assessed by immunofluorescence. ZO-1 protein (red), DAPI stain (blue), and merged ZO-1 protein and DAPI are presented. CON: pigs were fed with basal diet and challenged by sterile saline; SSY: pigs were fed with SeY-containing diet and challenged by sterile saline; DT: pigs were fed with basal diet and challenged by diquat; DSY: pigs were fed with SeY-containing diet and challenged by diquat.

As compared to the CON group, the villus height in the duodenum and the ratio of V/C in the duodenum, jejunum, and ileum were increased in the SSY group (Table 4 and Figure 2). However, the villus height in the jejunum and ileum was significantly decreased in the DT group (*P* < 0.05). The villus height and the ratio of V/C were higher in the DSY group than those in the DT group (*P* < 0.05). The distribution and abundance of the tight-junction protein ZO-1 are shown in Figure 3. As compared to the CON and DT groups, the localization of ZO-1 in the jejunum was enhanced in the SSY and DSY groups. The ZO-1 staining was diffused with little staining at the intercellular tight junction region in the DT group (indicating disruption of the tight junction).

### 3.4. Effect of SeY on Apoptosis Rate in Intestinal Epithelial Cell

**Figure 4:**
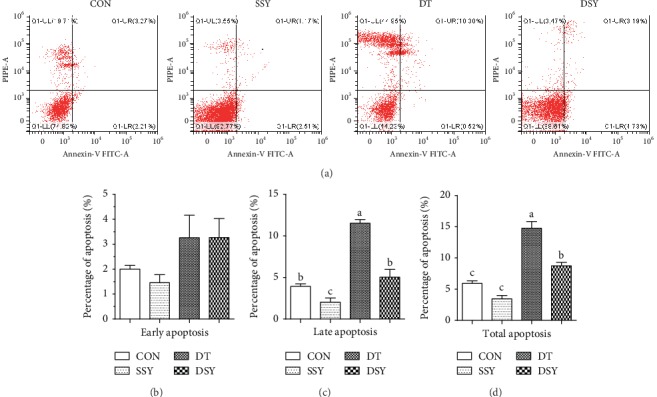
Effect of SeY on apoptosis rate in intestinal epithelial cells. (a) Evaluation of jejunal cell apoptosis by flow cytometry in weaned pigs exposed to SeY and diquat challenged. 30,000 cells were used in each acquisition reading. Frames were divided into 4 quadrants: Q1-UL represents necrotic cells; Q1-UR represents late apoptotic and early necrotic cells; Q1-LR represents early apoptotic cells; and Q1-LL represents normal cells; percentages of apoptotic cells of early apoptosis (b), late apoptosis (c), and total apoptosis (d) in the jejunum, respectively. ^a,b,c^Mean values with different letters on vertical bars indicate significant differences (*P* < 0.05). CON: pigs were fed with basal diet and challenged by sterile saline; SSY: pigs were fed with SeY-containing diet and challenged by sterile saline; DT: pigs were fed with basal diet and challenged by diquat; DSY: pigs were fed with SeY-containing diet and challenged by diquat.

The late and total apoptosis rates in the jejunal epithelial cells were significantly higher in the DT group than those in the CON group (Figure 4). As compared to the DT group, the late and total apoptosis rates of the jejunal epithelial cells were significantly decreased in the DSY group (*P* < 0.05).

### 3.5. Effect of SeY on Antioxidant Capacity in the Intestinal Mucosa

As compared to the CON group, the MDA content in the duodenum mucosa was significantly increased in the DT and DSY groups (Table 5). The CAT and T-AOC activities in the duodenum mucosa were higher in the DSY group than in the DT group (*P* < 0.05). Moreover, the GSH-Px and T-AOC activities in the jejunum and ileum mucosa were also higher in the DSY group than those in the DT group (*P* < 0.05).

### 3.6. Effect of SeY on Expressions of Critical Genes Related to Nutrient Absorption and Intestinal Barrier Functions

**Figure 5:**
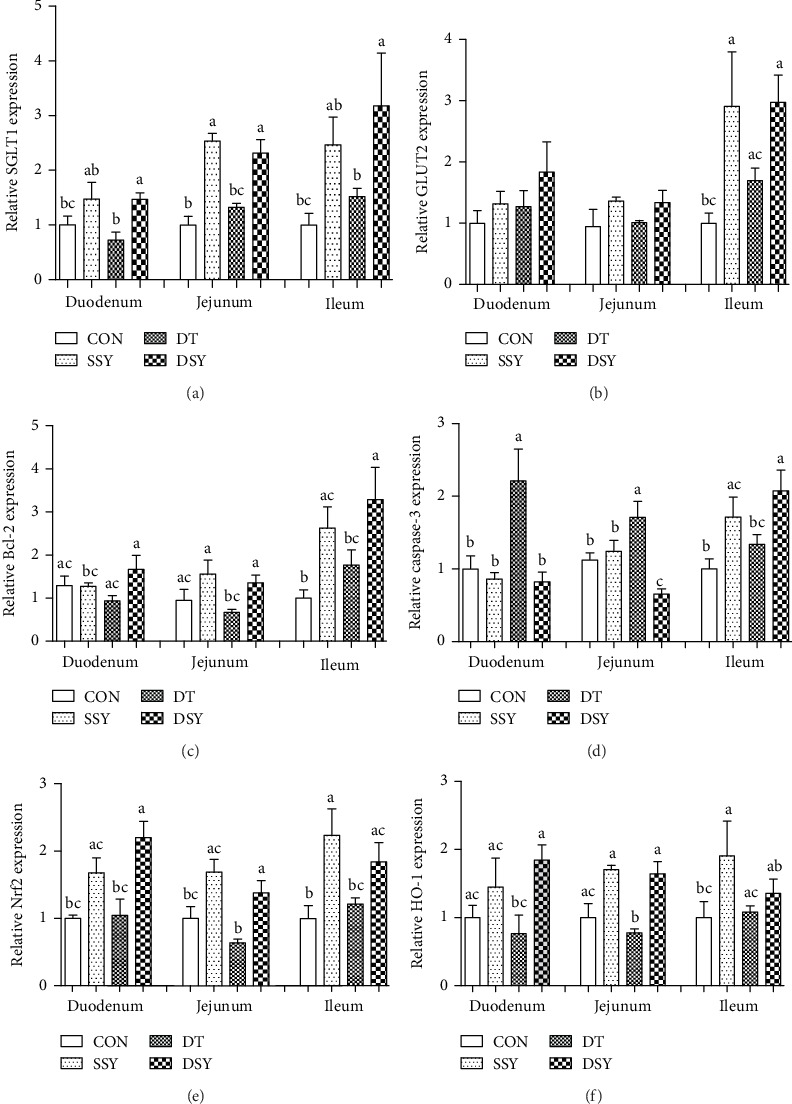
Relative expression levels of critical genes involved in the intestinal barrier functions. SGLT1: sodium glucose transport protein-1 (a); GLUT2: glucose transporter-2 (b); Bcl-2: B-cell lymphoma-2 (c); caspase-3: cysteine aspartic protease-3 (d); Nrf2: nuclear factor E2-related factor 2 (e); HO-1: heme oxygenase-1 (f). ^a,b,c^Mean values with different letters on vertical bars indicate significant differences (*P* < 0.05). CON: pigs were fed with basal diet and challenged by sterile saline; SSY: pigs were fed with SeY-containing diet and challenged by sterile saline; DT: pigs were fed with basal diet and challenged by diquat; DSY: pigs were fed with SeY-containing diet and challenged by diquat.

The expression levels of SGLT1 in the intestinal mucosa were higher in the DSY group than those in the DT group (*P* < 0.05; Figure 5). As compared to the CON group, SeY supplementation not only elevated the expression level of SGLT1 in the jejunum mucosa but also elevated the expression level of GLUT1 in the ileum mucosa (*P* < 0.05). Moreover, we found that SeY supplementation significantly elevated the expression levels of ZO-1 in the duodenum and jejunum mucosa in pigs upon oxidative stress (*P* < 0.05). As compared to the DT group, the expression levels of Bcl-1, Nrf-2, and HO-1 in the duodenum and jejunum mucosa were higher in the DSY group than those in the DT group (*P* < 0.05). In contrast, the expression levels of caspase-3 in the duodenum and jejunum mucosa were significantly decreased in the DSY group, as compared to those in the DT group (*P* < 0.05).

## 4. Discussion

Weaning is a critical developmental stage for mammals. At this stage, animals are more susceptible to exogenous stresses including the oxidative stress because of alterations of their living conditions [17, 18]. A previous study indicated that oxidative stress is one of the major contributors leading to growth retardation and intestinal mucosa disruption in neonatal animals including the pigs [11]. Selenium (Se) is an essential trace element for pigs. Importantly, Se is a critical component of the enzyme glutathione peroxidase (GSH-Px), which detoxifies lipid peroxides and provides protection of cellular and subcellular membranes against ROS damage [5]. In the present study, we investigated the effect of dietary SeY supplementation on growth performance and intestinal health in weaned pigs upon oxidative stress. The oxidative model was induced by using diquat injection, which is a well-established method [1, 13, 14]. We found that the ADG and ADFI were decreased in pigs upon diquat injection (DY and DSY). The result is consistent with a previous study on the weaned pigs that oxidative stress led to significant reduction of the growth performance [19]. However, SeY supplementation significantly attenuated the body-weight reduction in DYS after diquat injection. The result is consistent with previous studies on pigs and broilers that dietary SeY supplementation could improve their growth performance and elevate their antioxidative capacity [20, 21].

A previous study suggested that oxidative stress suppresses the intestinal barrier functions by inducing apoptosis of the intestinal epithelial cells [4]. DAO is a highly active intracellular enzyme in all mammalian intestinal mucosal epithelial cells, and the DAO activity in serum can act as a marker of intestinal epithelial cell maturity, integrity, and functional status [22]. D-lactic acid is a metabolite fermented by the gastrointestinal tract bacteria [23], and few of them can be absorbed into the blood under normal condition. However, a large amount of D-lactic acid can be absorbed into the blood circulation system through the impaired intestinal mucosa [24, 25]. In the present study, the serum concentrations of DAO and D-lactic acid were significantly higher in the DT group than those in the CON group, indicating disruption of the intestinal epithelial barrier upon diquat injection. However, both their concentrations were significantly decreased in the DSY group, as compared to those in the DT group.

An integrated intestinal morphological structure is of great importance for digestion and absorption [26]. In the present study, the villus height in the jejunum and ileum was decreased in the DT group, as compared to that in the CON group. This is consistent with a previous study that overproduction of ROS may lead to disruption of the intestinal morphology such as villus atrophy and crypt hyperplasia [27]. However, the villus height was higher in the DSY group than that in the DT group. Previous study indicated that the intestinal epithelial barrier is maintained and regulated by the tight junctions between the epithelial cells [28]. ZO-1 is one of the most important tight-junction proteins, which directly or indirectly anchors to the actin-based cytoskeleton and then forms a selective permeable barrier [29]. In the present study, the ZO-1 protein was highly expressed and localized in the apical intercellular region of the intestinal epithelium in the DSY group. Both these results suggested a protective effect of the SeY on the intestinal barrier in pigs upon oxidative stress.

Cell apoptosis is a physiological process that plays a vital role in maintaining the intestinal epithelial turnover [30]. However, excessive apoptosis disrupts the homeostasis of the intestinal epithelial cells, which may lead to mucosa atrophy and other intestinal disorders [31]. In the present study, the total apoptosis rate of the jejunal epithelial cells was higher in the diquat challenged pigs (DT and DSY) than that in the nonchallenged pigs (CON and SSY). The result is consistent with previous studies that overproduction of ROS can induce DNA damage and apoptosis in a variety of animal species and cell lines [32]. As compared to the DT group, the total apoptosis rate was significantly decreased in the DSY group. This may be attributed to the elevated antioxidative capacity in the intestinal mucosa [33]. A previous study indicated that diquat can directly catalyze the molecular oxygen to produce O^−2^ and H_2_O_2_, which initiates the lipid peroxidation and produces a large number of free radicals [34, 35]. Moreover, the SeY was reported to have the potentials to serve as a therapeutic antioxidant agent by reducing lipid peroxidation and ROS production, weakening DNA oxidative damage, and inhibiting cell apoptosis [8, 9]. In the present study, SeY supplementation significantly elevated the content of GSH-Px and CAT in the intestinal mucosa, which offers sufficient antioxidant molecules to abolish the free radicals.

We also explored the expression levels of several critical genes involved in the intestinal barrier functions. The SGLT1 is an active glucose transporter that takes up glucose into cells independent of the extracellular concentration of glucose. It plays a critical role in maintaining glucose homeostasis at both physiological and pathological levels [36]. In the present study, the expression level of the SGLT1 in the intestinal mucosa was higher in the DSY group than that in the DT group, indicating an improved absorption ability by SeY supplementation. The Bcl-2 and caspase-3 are two critical molecules involved in the regulation of apoptosis [37]. The Bcl-2 is a negative regulator of apoptosis, which can protect many types of cells from apoptosis [38], while the caspase-3 can act as one of the most important initiator caspases that are closely coupled to proapoptotic signals [39]. Once activated, initiation caspases cleave and activate downstream effector caspases (i.e., caspase-3), which in turn execute apoptosis by cleaving targeted cellular proteins [40]. In the diquat challenged pigs (DT and DSY), SeY supplementation significantly improved the expression level of Bcl-2 but downregulated the expression of caspase-3, which offers molecular basis for the decreased cell apoptosis in the jejunal epithelium. Moreover, the expression levels of two critical genes involved in the antioxidative signaling were determined. The Nrf2 is a critical transcription factor that can regulate genes involved in the production of a wide variety of antioxidant enzymes (i.e., glutathione and catalase) and detoxification or “stress-response” genes [41]. The HO-1 is one of the most important target genes of Nrf2, which can catalyze the rate-limiting step in heme degradation and produce free iron, biliverdin, and carbon monoxide. Importantly, the HO-1 has been implicated in the regulation of a series of biological processes including inflammation, apoptosis, fibrosis, and angiogenesis [42]. In the present study, their expression levels were higher in the DSY group than those in the DT group. The result is in accordance with the measurements of the antioxidative enzymes in the intestinal mucosa. Both results indicated that the SeY can act as a potential therapeutic antioxidant agent.

## 5. Conclusions

The present study suggested that dietary SeY supplementation can attenuate the growth retardation of the weaned pigs in response to the oxidative stress. Moreover, SeY alleviates the oxidative stress-induced disruption of the intestinal mucosa, which was associated with elevated mucosal antioxidative capacity and improved intestinal barrier functions. The beneficial effects of SeY supplementation on the growth and intestinal health upon oxidative stress suggested that it can serve as a potential therapeutic antioxidant agent.

## Figures and Tables

**Table 1:** Composition and nutrient level of experimental diet.

Ingredients	%	Nutrient level	Contents
Corn	28.31	Digestible energy (calculated, MJ/kg)	14.78
Extruded corn	24.87	Crude protein (%)	19.68
Soybean meal	8.50	Calcium (%)	0.81
Extruded full-fat soybean	10.30	Available phosphorus (%)	0.55
Fish meal	4.20	Lysine	1.35
Whey powder	7.00	Methionine	0.42
Soybean protein concentrate	8.00	Methionine+cysteine	0.60
Soybean oil	2.00	Threonine	0.79
Sucrose	4.00	Tryptophan	0.22
Limestone	0.90		
Dicalcium phosphate	0.50		
NaCl	0.30		
L-Lysine HCl (78%)	0.47		
DL-methionine	0.15		
L-Threonine (98.5%)	0.13		
Tryptophan (98%)	0.03		
Chloride choline	0.10		
Vitamin premix^1^	0.04		
Mineral premix^2^	0.20		
Total	100		

^1^The vitamin premix provided the following per kg of diet: 9000 IU of VA, 3000 IU of VD 3, 20 IU of VE, 3 mg of VK 3, 1.5 mg of VB1, 4 mg of VB 2, 3 mg of VB6, 0.02 mg of VB12, 30 mg of niacin, 15 mg of pantothenic acid, 0.75 mg of folic acid, and 0.1 mg of biotin. ^2^The mineral premix provided the following per kg of diet: 100 mg Fe, 6 mg Cu, 100 mg Zn, 4 mg Mn, and 0.30 mg I.

**Table 2:** Primer sequences used for quantitative RT-PCR.

Gene	Accession no.	Primer sequences (5′-3′)	Product length (bp)
Nrf2	XM_003133500.5	GCCCCTGGAAGCGTTAAAC	67
		GGACTGTATCCCCAGAAGGTTGT	

HO-1	NM_001004027	CGCTCCCGAATGAACAC	112
		GCTCCTGCACCTCCTC	

Bcl-2	XM_021099593.1	GCTACTTACTGCCAAAGGGA	110
		TTCAGGCGGAGCTGTAAGAG	

Caspase-3	NM_214131.1	GGAATGGCATGTCGATCTGGT	105
		ACTGTCCGTCTCAATCCCA	

SGLT1	NM_001164021.1	CGCGTCCGGTGTGAAAG	137
		CTTCCCGATATCTACACATTCCA	

GLUT2	NM_001097417.1	GAGGCAGCAGTAGGGAATCTTCGAGCA	140
		ACAGTTACTCTGACACCCGTTCTTC	

*β*-Actin	XM_003124280.5	TGGAA CGGTG AAGGT GACAGC	177
		GCTTTTGGGAAGGCAGGGACT	

Nrf2: nuclear factor E2-related factor 2; HO-1: heme oxygenase-1; Bcl-2: B-cell lymphoma-2; caspase-3: cysteine aspartic protease-3; SGLT1: sodium glucose transport protein-1; GLUT2: glucose transporter-2.

**Table 3:** Effect of SeY on growth performance in weaned pigs upon oxidative stress.

Items	Treatments				*P* value			
	CON	SSY	DT	DSY	SEM	SeY	Diquat	SeY∗diquat
Prechallenged (1–15 d)								
ADG (g/day)	340	320	300	320	10			
ADFI (g/day)	410.23	396.90	374.89	38.30	14.62			
F : G	1.20	1.24	1.22	1.21	0.08			
Postchallenged (16–21 d)								
ADG (g/day)	438.33^a^	452.50^a^	-95.83^c^	164.17^b^	63.31	0.03	<0.01	0.048
ADFI (g/day)	511.46^a^	505.25^a^	195.21^c^	311.67^b^	31.69	0.03	<0.01	0.039
F : G	1.17	1.12	—	1.90				

^1^Values are means ± SEM (*n* = 6), nonchallenged pigs (CON, fed with basal diet), diquat-challenged pigs (DT, fed with basal diet), and SeY-treated pigs (fed with basal diet containing 250 mg/kg SeY) challenged by sterile saline (SSY) or diquat (DSY). ^2a,b,c^Mean values within a row with unlike superscript letters were significantly different (*P* < 0.05). ^3^ADFI = average daily feed intake; ADG = average daily gain; G/F = the ratio of gain to feed intake.

**Table 4:** Effect of SeY on intestinal morphology in weaned pigs upon oxidative stress.

Items	Treatments					*P* value		
	CON	SSY	DT	DSY	SEM	SeY	Diquat	SeY × diquat
Duodenum								
Villus height (mm)	201.59^b^	228.13^a^	185.31^b^	203.81^b^	26.41	0.08	0.11	0.74
Crypt depth (mm)	87.65	80.39	93.79	83.71	13.23	0.12	0.75	0.87
V/C	2.19 ^c^	2.92 ^a^	1.99^bc^	2.46^ac^	0.5	0.01	0.11	0.52
Jejunum								
Villus height (mm)	144.27^ab^	181.36^a^	124.18^c^	157.89^a^	5.98	<0.01	0.03	0.84
Crypt depth (mm)	67.73	55.83	67.88	63.81	2.62	0.14	0.44	0.46
V/C	2.15^bc^	3.27^a^	1.87^c^	2.57^b^	0.15	<0.01	0.02	0.28
Ileum								
Villus height (mm)	155.54^a^	196.36^a^	142.29^b^	175.86^a^	8.47	0.18	0.15	0.51
Crypt depth (mm)	66.49^ab^	59.11^b^	75.34^a^	56.38^b^	2.59	<0.01	0.45	0.51
V/C	2.29^bc^	3.21^a^	1.91^b^	2.66^ac^	0.15	<0.01	0.01	0.61

^1^Values are means ± SEM (*n* = 6), nonchallenged pigs (CON, fed with basal diet), diquat-challenged pigs (DT, fed with basal diet), and SeY-treated pigs (fed with basal diet containing 250 mg/kg SeY) challenged by sterile saline (SSY) or diquat (DSY). ^2a,b,c^Mean values within a row with unlike superscript letters were significantly different (*P* < 0.05).

**Table 5:** Effect of SeY on antioxidant capacity of the intestinal mucosa.

Items	Treatments					*P* value		
	CON	SSY	DT	DSY	SEM	SeY	Diquat	SeY × diquat
Duodenum								
GSH-Px (mg/gprot)	56.61	58.61	66.28	78.6	2.66	0.46	0.03	0.73
T-AOC (U/mgprot)	0.49^ac^	0.78^ac^	0.45^bc^	0.92^a^	0.37	0.02	0.76	0.54
MDA (nmol/mL)	0.23^c^	0.43^bc^	0.92^a^	0.62^b^	0.07	0.59	<0.01	0.03
CAT (U/gprot)	15.50^b^	20.02^b^	20.42^b^	30.78^a^	1.84	0.02	0.02	0.32
Jejunum								
GSH-Px (mg/gprot)	95.36^a^	92.79^a^	72.41^b^	97.41^a^	3.39	0.09	0.09	0.04
T-AOC (U/mgprot)	1.50^ac^	1.33^a^	0.80^bc^	1.30^a^	0.39	0.23	0.02	0.02
MDA (nmol/mL)	1.87^a^	1.43^ac^	1.78^a^	1.14^b^	0.47	0.04	0.83	0.40
CAT (U/gprot)	33.5	28.52	30.56	33.34	1.32	0.69	0.73	0.17
Ileum								
GSH-Px (mg/gprot)	76.74^ab^	85.00^a^	71.13^b^	95.49^a^	3.64	0.02	0.71	0.23
T-AOC (U/mgprot)	0.24^bc^	0.29^ac^	0.22^bc^	0.41^a^	0.13	0.04	0.35	0.19
MDA (nmol/mL)	0.71^a^	0.59^b^	1.38^a^	0.57^b^	0.58	0.09	0.34	0.03
CAT (U/gprot)	8.58^b^	8.24^b^	6.40^b^	12.17^a^	0.59	<0.01	0.25	<0.01

^1^Values are means ± SEM (*n* = 6), nonchallenged pigs (CON, fed with basal diet), diquat-challenged pigs (DT, fed with basal diet), and SeY-treated pigs (fed with basal diet containing 250 mg/kg SeY) challenged by sterile saline (SSY) or diquat (DSY). ^2a,b,c^Mean values within a row with unlike superscript letters were significantly different (*P* < 0.05). ^3^GSH-Px: glutathione peroxidase; T-AOC: total antioxidant capacity; MDA: malondialdehyde; CAT: catalase.

## Data Availability

The data used to support the findings of this study are available from the corresponding author upon request.
